# Gut Microbiome and Plasma Metabolome Signatures in Middle-Aged Mice With Cognitive Dysfunction Induced by Chronic Neuropathic Pain

**DOI:** 10.3389/fnmol.2021.806700

**Published:** 2022-01-04

**Authors:** Dongyu Hua, Shan Li, Shiyong Li, Xuan Wang, Yue Wang, Zheng Xie, Yilin Zhao, Jie Zhang, Ailin Luo

**Affiliations:** Department of Anesthesiology, Tongji Hospital, Tongji Medical College, Huazhong University of Science and Technology, Wuhan, China

**Keywords:** pain, cognitive dysfunction, gut microbiome, metabolites, endocannabinoids

## Abstract

Patients with chronic neuropathic pain (CNP) often complain about their terrible memory, especially the speed of information processing. Accumulating evidence suggests a possible link between gut microbiota and pain processing as well as cognitive function *via* the microbiota-gut-brain axis. This study aimed at exploring the fecal microbiome and plasma metabolite profiles in middle-aged spared nerve injury (SNI) mice model with cognitive dysfunction (CD) induced by CNP. The hierarchical cluster analysis of performance in the Morris water maze test was used to classify SNI mice with CD or without CD [i.e., non-CD (NCD)] phenotype. 16S rRNA sequencing revealed a lower diversity of gut bacteria in SNI mice, and the increase of *Actinobacteria, Proteus*, and *Bifidobacterium* might contribute to the cognitive impairment in the CNP condition. The plasma metabolome analysis showed that the endocannabinoid (eCB) system, disturbances of lipids, and amino acid metabolism might be the dominant signatures of CD mice. The fecal microbiota transplantation of the Sham (not CD) group improved allodynia and cognitive performance in pseudo-germ-free mice *via* normalizing the mRNA expression of eCB receptors, such as *cn1r, cn2r*, and *htr1a*, reflecting the effects of gut bacteria on metabolic activity. Collectively, the findings of this study suggest that the modulation of gut microbiota and eCB signaling may serve as therapeutic targets for cognitive deficits in patients with CNP.

## Introduction

Accumulating preclinical and clinical evidence suggests that cognitive impairment is a common comorbidity of chronic neuropathic pain (CNP) (Attal et al., 2014; Mazza et al., 2018; Fonseca-Rodrigues et al., 2021; Rouch et al., 2021). On the one hand, neuropathic pain results in worse cognitive performance, whereas on the other hand, cognitive deficits also affect the perception of pain (Fonseca-Rodrigues et al., 2021). Previous studies have proposed several potential mechanisms about the comorbidity, such as limited attention capacity, brain structural changes, altered neurotransmitters, receptors, and neural mediators (e.g., enzymes, neurotrophic factors, and cytokines) (Moriarty et al., 2011; Mazza et al., 2018). However, the mechanisms underlying cognitive impairment associated with CNP have not yet been elucidated. Considering the fact that CNP and cognitive impairment are refractory to current pharmacological agents, specific targets for indicating or preventing the comorbidity are urgently needed.

Several studies have proposed that gut microbiota play critical roles in several central nervous system (CNS)-related conditions, such as pain and cognition (Amaral et al., 2008; Cryan and Dinan, 2012; Shen et al., 2017; Guo et al., 2019). Recent evidence suggests that dysbiosis exists in several types of CNP and cognitive impairments (Jiang et al., 2017; Lin et al., 2020; Marttinen et al., 2020)^.^ Moreover, the interventions of gut microbiota, such as probiotics and fecal microbiota transplantation (FMT), showed beneficial effects on the two disorders (Van Laar et al., 2019; Bonomo et al., 2020; Cuozzo et al., 2021). Studies have also revealed that gut microbiota regulates the development of CNP or cognitive impairment through the vagus nerve, endocrine, and metabolic as well as immune communications (Sampson and Mazmanian, 2015; Sun et al., 2020; Chen et al., 2021). However, it is not known how the microbiota affects the comorbidity.

Interactions between the host and microbiota cause a wide fluctuation in the circulating neuroactive substances, such as neurotransmitters, short-chain fatty acids, and bile acids (Cryan and Dinan, 2012; Sharon et al., 2016; Li et al., 2020). It is worth noting that the disturbance of the circulating metabolites is one of the major routes through which microbiota affect the brain. Therefore, this calls for the combinative analyses of the microbiome and plasma metabolome, which will confirm the microbial signatures and related neural pathways. In this study, mice with cognitive impairment phenotype were screened in a spared nerve injury (SNI) CNP model based on the behavioral results. We explored the alterations of gut microbiota and plasma metabolites by performing 16S rRNA sequencing and non-targeted metabolomics, which is the overarching goal of identifying potential biomarkers for predicting the comorbidity of CNP and cognitive impairment. Furthermore, the relationship between abnormal bacteria and metabolites was analyzed to evaluate the possible metabolic pathways involved in the comorbidity. Finally, pseudo-germ-free (pGF) mice were constructed to investigate the effects of FMT on endocannabinoid (eCB) signaling and pain as well as cognition.

## Materials and Methods

### Animals

Of note, 2- or 10-month-old male C57BL/6 mice in this study were obtained from the Animal Center of Tongji Hospital. In total, 71 mice were enrolled and randomly divided into different groups. Five mice were housed in an individual ventilated cage at 22 ± 2°C and 12-h light/dark cycles (lights on 8:00 a.m.) and with *ad libitum* food and water. Animals were acclimated to the environmental conditions for 7 days before the experiment. Procedures for the animal experiments were in accordance with the ethical guidelines of the National Institutes of Health and the International Association for the Study of Pain. This study was approved by the Experimental Animal Committee of Tongji Hospital, Tongji Medical College, Huazhong University of Science and Technology (Wuhan, China). Notably, efforts were made to minimize animal suffering and the number of animals used.

### Establishment of SNI Mice Model

A previous study found that middle-aged (i.e., 10 months old) rodents were more susceptible to cognitive impairment related to chronic pain compared to young (i.e., 3 months old) and old (i.e., 22 months old) groups (Leite-Almeida et al., 2009). Thus, middle-aged mice were used to establish the cognitive impairment model induced by CNP in this study. Mice were anesthetized with 3% isoflurane for induction and 1.5% isoflurane for maintenance. After dissecting the skin and the musculature in the surgical site on the left limb lateral surface, the sciatic nerve and its three terminal branches (i.e., sural, common peroneal, and tibial nerves) were exposed. Then, the common peroneal and tibial nerves were tightly ligated with 4–0 silk ligatures and dissected the distal to the ligation. The mice were placed on a heating blanket to keep warm during the procedure. After suturing the skin and muscles, the mice were returned to their previous cages. Mice in the Sham group also underwent similar procedures, but the nerves were only exposed without touching.

### Open Field Test

The open field test (OFT) was used to test the effect of SNI surgery on motor activities. After being habituated to the environment for 30 min, animals were placed at the center of the Plexiglas chamber (40 × 40 × 40 cm) to explore freely for 5 min, and the total distance traveled (in centimeters) was automatically monitored and analyzed using the automatic tracking system (ZhongShi technology, Beijing, China). The inner floor and wall of the chamber were cleaned with 3% acetic acid in the interval.

### Mechanical Withdraw Threshold

The mechanical allodynic response was used as a hall marker for CNP in SNI mice. Mice were placed in separate Plexiglas light-transparent chambers on the elevated mesh wire for 30 min before the test to habituate to the examination condition. The von Frey filaments (0.008, 0.02, 0.04, 0.07, 0.16, 0.4, 0.6, and 1 g) were applied to the lateral surface (sural nerve territory) on the left foot for 6 s and repeated five consecutive times. The procedure started with the lowest force (0.008 g) and progressively increased to higher von Frey filaments until positive responses occurred in the experiment animal. Notably, quick paw withdrawal or licking was considered a positive pain-like response. The next lower filament was applied when a positive response was observed at least three times in five consecutive times under the same force. It should be noted that the next higher filament was also used when no positive response occurred. The lowest filament (in grams) that can induce a positive response was recorded as the MWT.

### Morris Water Maze Test

The *Morris water maze test* (MWMT), consisting of a 5-day training and a probe test on the 6th day, was performed to evaluate the spatial reference memory according to our previous studies (Zhan et al., 2018, 2019). Mice were trained to locate the 10-cm-diameter hidden platform (submerged 0.5–1 cm below the water surface) three times per day for 5 consecutive days in a circular pool (diameter 120 cm, height 50 cm) filled with opaque water (23 ± 1°C). After locating the hidden platform, mice were allowed to stay there for 15 s. Mice that failed to find the hidden platform within 60 s were gently guided to the platform and allowed to remain there for 15 s. The platform was placed at the same location throughout the 5-day training test. The time taken to locate the platform (escape latency) was recorded using a digital camera (ZhongShi Technology, Beijing, China) and used to evaluate the spatial learning ability of the mice. A probe test was then performed on the 6th day to evaluate the spatial memory capacity of the animal. After the 5-day training, the hidden platform was removed from the pool, and mice were given 60 s to swim freely in the pool. The number of crossing times and time spent in the target quadrant were automatically recorded.

### 16S rRNA Sequencing of Fecal Samples

Total fecal genomic DNA was extracted using a DNA Extraction Kit (Qiagen Inc., Valencia, CA, USA) according to the instructions of the manufacturer. The concentration of DNA was then determined using NanoDrop (NanoDrop Technologies Inc., DE, USA) and agarose gel electrophoresis. The extracted genomic DNA was used as a template for PCR amplification using the barcoded primers and Tks Gflex DNA Polymerase (Takara Biomedicals, Beijing, China). For the bacterial diversity analysis, V3–V4 variable regions of 16S rRNA genes were amplified using universal primers: 343 F (5′-TACGGRAGGCAGCAG-3′) and 798 R (5′-AGGGTATCTAATCCT-3′). Raw sequencing data were then spliced using the sliding window trimming approach to cut off ambiguous bases and low-quality sequences. Then, further denoising was performed on the sequences using the QIIME software, and reads with 75% of bases above Q20 were retained. Finally, operational taxonomic units and clustering were performed using the VSEARCH software, with 97% similarity as the cutoff.

### Liquid Chromatography-Mass Spectrometry Analysis of Plasma Metabolites

Plasma samples, which had been stored at −80°C, were thawed in an ice bath before the process. The metabolites were extracted as previously described (Braundmeier-Fleming et al., 2016; Song et al., 2021). In brief, 20 μl of the sample was dissolved in an extraction solution consisting of 2-chloro-l-phenylalanine, methanol, and acetonitrile, followed by centrifugation at 13,000 rpm for 15 min at 4°C. The supernatant was then transferred into a brown glass vial and dried in a freeze concentration centrifugal dryer. Each sample was homogenized using a mixture of methanol and water (1/4 v/v) and centrifuged at 13,000 rpm for 5 min at 4°C. The supernatants were collected in glass vials and stored at −80°C until the liquid chromatography-mass spectrometry (LC-MS) analysis. After sample preparation, plasma metabolites were identified and quantified by ACQUITY UPLC I-Class system (Waters Corporation, Milford, CT, USA) and VION IMS QTOF Mass Spectrometer (Waters Corporation, Milford, CT, USA) in both positive and negative ion modes. Gradient elution (mobile A: water with 0.1% formic acid; mobile B: Acetonitrile/Methanol with 0.1% formic acid) was performed to separate the samples on an ACE C18 column (1.7 μm, 2.1 × 100 mm) at a flow rate of 0.4 ml/min. The linear gradient was as follows: 0 min, 1% B; 1 min, 30% B; 2.5 min, 60% B; 6.5 min, 90% B; 8.5 min, 100% B; 10.7 min, 100% B; 10.8 min, 1% B; and 13 min, 1% B. Finally, the acquired raw data were analyzed using the progenesis QI software (Waters Corporation, Milford, CT, USA).

### pGF Mice Modeling

The pGF mice were established according to our previous studies (Zhan et al., 2018; Yang et al., 2019). In brief, broad-spectrum antibiotics were dissolved in drinking water in the following concentrations: 1 g/L ampicillin, 1 g/L neomycin sulfate, and 1 g/L metronidazole (Sigma–Aldrich, Shanghai, China). Mice were exposed to plain water or water with antibiotics for 14 days. Notably, the drinking solution was replenished every 2 days.

### Fecal Microbiota Transplantation

Fresh feces were collected from anal orifices of donors and immediately used to prepare the microbiota solution. The solution was prepared by diluting 1 g of fresh feces into 10 ml sterile phosphate-buffered saline (PBS), followed by a vortex to suspend the microbiota. After antibiotics treatment, pGF mice were given 0.2 ml PBS or fecal microbiota suspension obtained from the Sham and cognitive dysfunction (CD) group mice by oral gavage once a day for 14 consecutive days.

### Measurement of eCB Signaling in the Prefrontal Cortex Tissues Using Quantitative PCR

Total RNA was extracted from prefrontal cortex (PFC) tissues using TRIzol reagent (Takara Biomedicals, Beijing, China) in accordance with the instructions of the manufacturer. In brief, tissues were immersed in 500 μl TRIzol reagent and then homogenized on ice with a homogenizer. Then, chloroform (100 μl) was added to the suspension, followed by mixing and centrifugation to isolate the aqueous phase with RNA. Subsequently, RNA was precipitated with a volume of isopropyl alcohol and washed with 75% ethanol. The concentration and purity of RNA suspended with diethylpyrocarbonate (DEPC)-treated water was evaluated using a NanoDrop (NanoDrop Technologies Inc., DE, USA) according to the absorbance at 260 (*A260*) and 280 nm (*A280*). Notably, each sample was measured three times to ensure the precision of measurements. Then, cDNA was synthesized from 1 μg of RNA using a cDNA Synthesis Kit (Vazyme, Nanjing, China) according to the protocol of the manufacture and then used for the quantitative PCR (qPCR) analysis. Primers of the target gene for the eCB system were designed using Primer-Blast and are listed in Supplementary Table 1. Each sample was run in duplicate in a 20-μl reaction, including 0.5 μl forward and reverse primers, 20 ng of cDNA, and 10 μl SYBR Green Master Mix (Vazyme, Nanjing, China). The following reaction conditions were used for RNA amplification: 95°C for 5 min, followed by 45 cycles of denaturing 20 s at 95°C, annealing 30″ at 70°C, and extension 30″ at 72°C. Relative gene expression was normalized to that of glyceraldehyde-3-phosphate dehydrogenase (GAPDH) mRNA levels and quantified using the ΔΔCt method. The mean relative expression level of each gene in the control group was standardized to 1 to facilitate the comparison. The results were presented as fold change relative to the control group.

### Statistical Analysis

All analyses were performed using GraphPad Prism software version 8.0, and all data were presented as the mean ± SEM. Comparisons among groups were performed using the one-way ANOVA or two-way ANOVA, followed by Tukey's *post hoc* test. For different time points, two-way repeated-measures (RM) ANOVA was performed to compare the difference among groups. Discontinuous data, such as the platform crossing times of the animal in the probe test, and sequencing data not conforming to the normal distribution were analyzed using Kruskal–Wallis non-parametric test, followed by Dunn's *post hoc* test. In hierarchical cluster analysis, the data were first standardized using *z*-scores. Then, the hierarchical cluster analysis of escape latency, platform crossing times, and time spent in the target quadrant was performed using Ward's method. Squared Euclidean distance was applied as the distance measure, and mice were classified as CD or non-CD (NCD) clusters. Finally, Spearman's correlation was performed to analyze the correlation between gut microbiota and plasma metabolites. *P* < 0.05 was considered statistically significant.

## Results

### SNI-Induced CNP Impaired Spatial Reference Memory in Middle-Aged Mice

**Figure 1:**
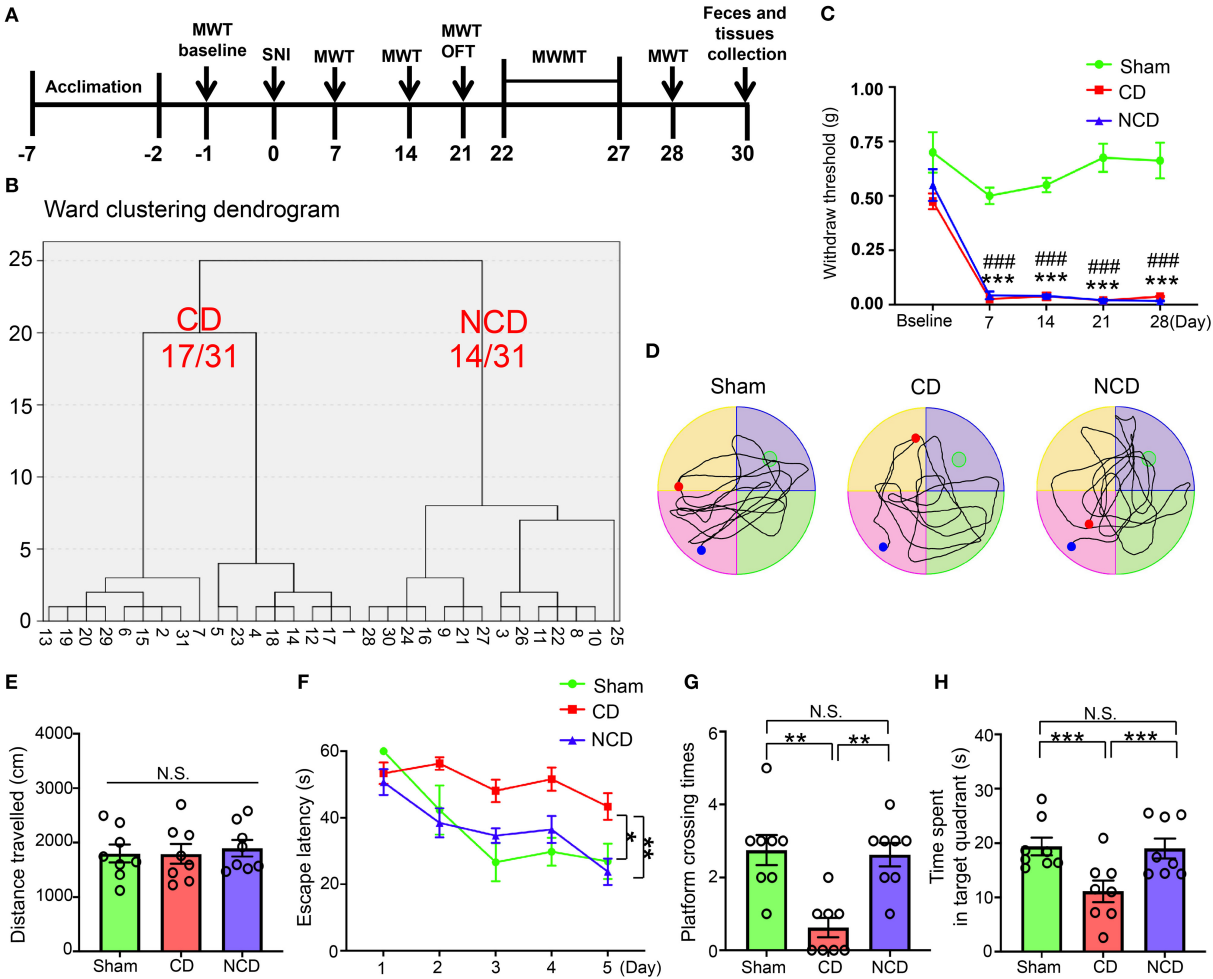
Spatial learning and reference memory were impaired in CD mice. **(A)** The schedule of the experiment. SNI was performed on day 0 after acclimation. MWT was measured on day 1 before the surgery and on day 7, 14, 21, and 28 after SNI. From day 22–27 after the surgery, MWMT was applied to evaluate the spatial reference memory of animals in Sham and SNI groups. Tissues and fecal samples were collected on day 30 post-SNI. **(B)** MWT [two-way repeated-measures (RM) ANOVA; Time: *F*_(4, 84)_ = 39.63, *P* < 0.001; Group: *F*_(2, 21)_ = 197.9, *P* < 0.001; Interaction: *F*_(8, 84)_ = 6.184, *P* < 0.001]. **(C)** Dendrogram of hierarchical clustering analysis. A total of 31 mice after SNI were divided into CD and NCD groups by the escape latency, platform crossing, and time spent in the target quadrant results of hierarchical clustering analysis. Of note, 17 of 31 mice (CD group) exhibited worse performance in the spatial learning and memory functions, including the latency to reach the target platform, the times of crossing the platform, and the time spent in the target quadrant. **(D)** The graphs of the representative traces of Sham, CD, and NCD groups in the probe test. **(E)** Total distance in OFT [one-way ANOVA; *F*_(2, 21)_ = 0.124, *P* > 0.05]. **(F)** The latency to reach the submerged platform in MWMT [two-way RM ANOVA; Time: *F*_(4, 84)_ = 12.38, *P* < 0.001; Group: *F*_(2, 21)_ = 43.37, *P* < 0.001; Interaction: *F*_(8, 84)_ = 1.992, *P* = 0.057]. **(G)** Crossing times in the probe test [Kruskal–Wallis non-parametric test; *H*_(2, 21)_ = 12.94; *P* = 0.002]. **(H)** The time spent in the target quadrant [one-way ANOVA; *F*_(2, 21)_ = 6.5, *P* = 0.006]. Data are presented as means ± SEM (*n* = 8 per group) and analyzed by one-way ANOVA or two-way ANOVA followed by Tukey's *post hoc* test or Kruskal–Wallis non-parametric test followed by Dunn's *post hoc* test. *P < 0.05, **P < 0.01, ***P < 0.001, CD vs. Sham. ^**###**^*P* < 0.001, NCD vs. Sham. CD, cognitive dysfunction; SNI, spare nerve injury; MWMT, Morris water maze test; MWT, mechanical withdraw threshold; NCD, non-cognitive dysfunction; N.S., not significant; OFT, open field test.

A total of 31 mice underwent SNI to develop neuropathic pain (Figure 1A). Mechanical allodynia was observed on the 7th post-SNI and persisted until the end of the experiment (day 28) (Figure 1C). After 1 month, SNI mice were divided into CD (54.8%) group or NCD (45.2%) group according to the hierarchical clustering analysis of the MWMT performance (Figure 1B). The representative trace graphs of the three groups in the probe trial were presented in Figure 1D. OFT was performed before MWMT to evaluate the locomotor activity, and the total distance traveled in the open field showed no significant difference among the groups, suggesting that the locomotor ability was not compromised by SNI (Figure 1E). In addition, MWMT was used to evaluate reference memory among the groups. Results showed that the CD mice exhibited significantly higher escape latency in the training test compared to the Sham and NCD mice, indicating the impairment learning capacity (Figure 1F). In the probe trial, the time spent in the target quadrant and the numbers of platform crossing were significantly decreased in the CD mice compared to the Sham and NCD mice (Figures 1G,H). These results suggest that the hierarchical clustering analysis is an effective approach to discriminate CD mice after SNI surgery. Excluding the mice without cognitive impairment from the SNI group facilitates us to find specific associations with CNP-related CD (in this case, gut microbiota and plasma metabolites).

### Differential Gut Microbiota Profiling Among the Sham, CD, and NCD Mice

**Figure 2:**
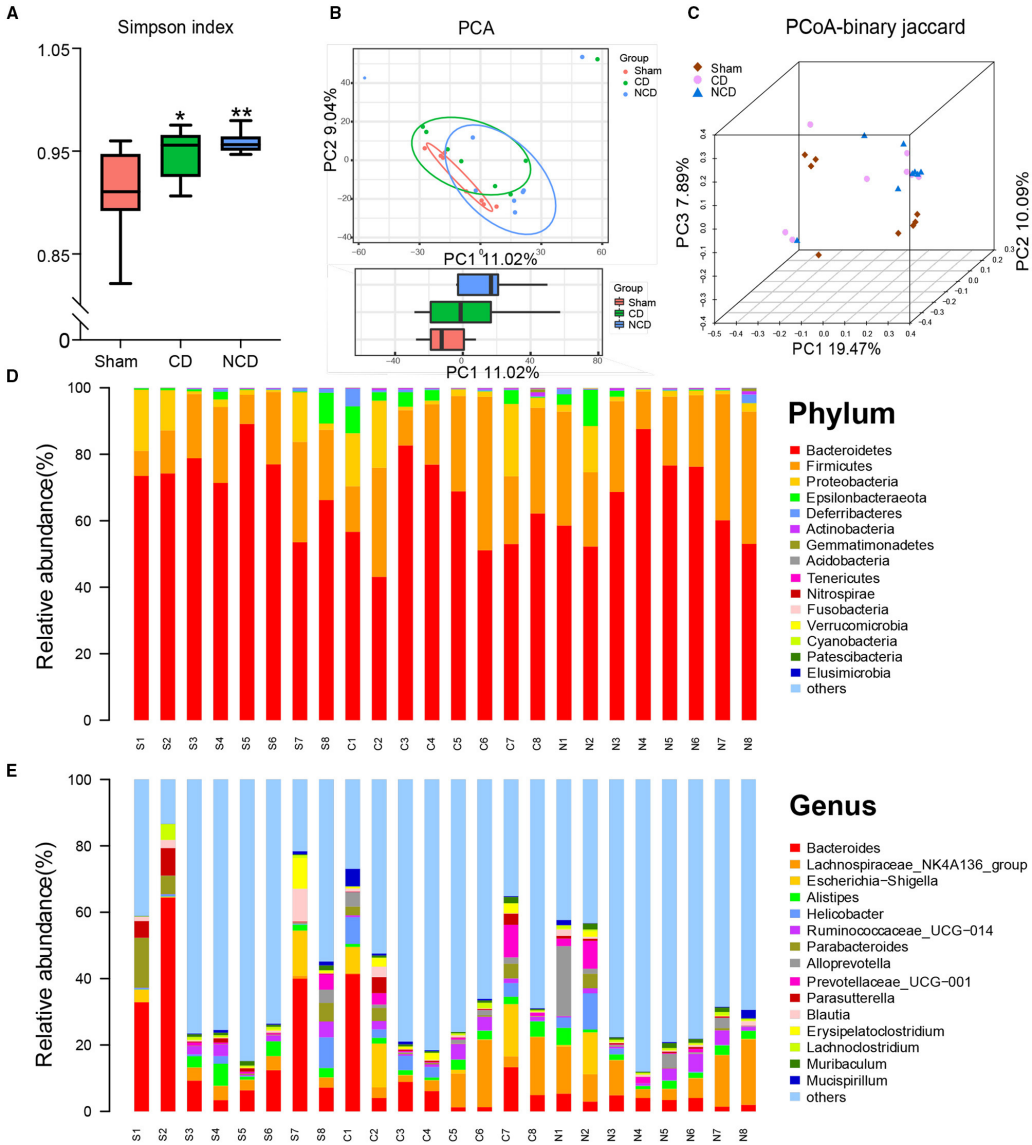
Differential profiles of gut microbiota among Sham, CD, and NCD groups. **(A)** Simpson index [*F*_(2, 21)_ = 6.557, *P* = 0.006]. **(B)** The PCA of gut microbiota (PC1 vs. PC2). **(C)** PCoA-binary Jaccard dissimilarity. **(D)** The top 15 abundant flora among the groups at the phylum level. **(E)** The top 15 abundant flora among the groups at the genus level. Data are presented as means ± SEM (*n* = 8 per group) and analyzed by one-way ANOVA followed by Tukey's *post hoc* test. *P < 0.05, **P < 0.01. CD, cognitive dysfunction; NCD, non-cognitive dysfunction; N.S., not significant.

**Figure 3:**
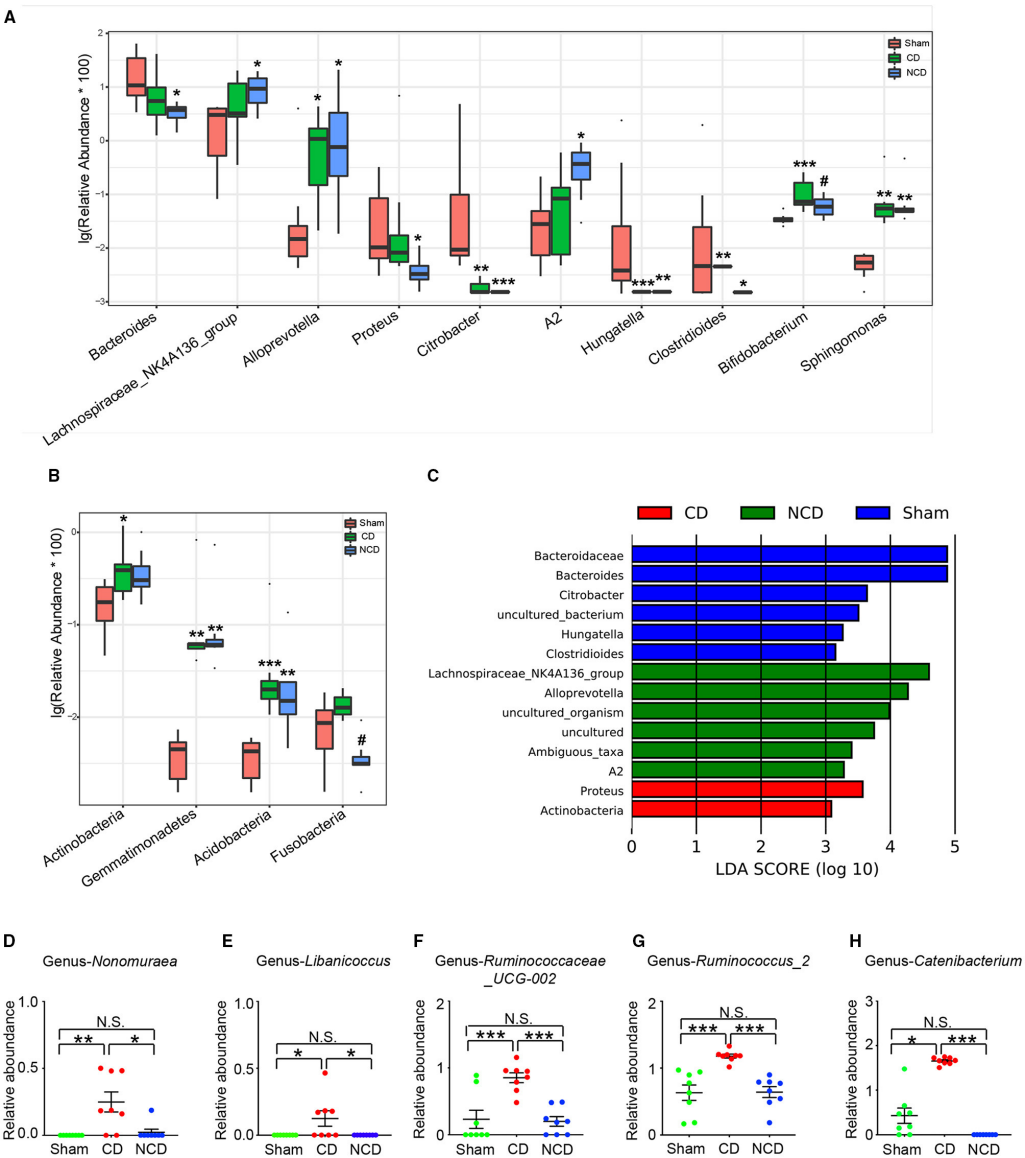
Microbiota with significant changes at phylum and genus levels among the groups. **(A)** The top 10 flora with significant difference among the groups at genus level: *Bacteroides* [Kruskal–Wallis non-parametric test, *H*_(2, 21)_ = 8.205; *P* = 0.02]; *Lachnospiraceae-NK4A136-group* [Kruskal–Wallis non-parametric test, *H*_(2, 21)_ = 6.14; *P* = 0.046]; *Alloprevotella* [Kruskal–Wallis non-parametric test, *H*_(2, 21)_ = 8.315; *P* = 0.02]; *Proteus* [Kruskal–Wallis non-parametric test, *H*_(2, 21)_ = 8.05; *P* = 0.02]; *Citrobacter* [Kruskal–Wallis non-parametric test, *H*_(2, 21)_ = 17.92; *P* = 0.0001]; *A2* [Kruskal–Wallis non-parametric test, *H*_(2, 21)_ = 7.985; *P* = 0.02]; *Hungatella* [Kruskal–Wallis non-parametric test, *H*_(2, 21)_ = 15.91; *P* = 0.0004]; *Clostridioides* [Kruskal–Wallis non-parametric test, *H*_(2, 21)_ = 10.64; *P* = 0.005]; *Bifidobacterium* [Kruskal–Wallis non-parametric test, *H*_(2, 21)_ = 11.33; *P* = 0.0035]; *Sphingomonas* [Kruskal–Wallis non-parametric test, *H*_(2, 21)_ = 15.44; *P* = 0.0004]. **(B)** The phylum with significant difference among the groups: *Actinobacteria* [Kruskal–Wallis non-parametric test, *H*_(2, 21)_ = 6.995; *P* = 0.0008]; *Gemmatimonadetes* [Kruskal–Wallis non-parametric test, *H*_(2, 21)_ = 15.38; *P* = 0.0005]; *Acidobacteria* [Kruskal–Wallis non-parametric test, *H*_(2, 21)_ = 14.41; *P* = 0.0007]; *Fusobacteria* [Kruskal–Wallis non-parametric test, *H*_(2, 21)_ = 12.56; *P* = 0.002]. **(C)** Taxonomic groups showing linear discriminant analysis (LDA) scores > 3.0. **(D)** Significant altered gut bacteria in CD mice compared with Sham and NCD mice. *Nonomuraea* [Kruskal–Wallis non-parametric test, *H*_(2, 21)_ = 11.77; *P* = 0.003]; *Libanicoccus* [Kruskal–Wallis non-parametric test, *H*_(2, 21)_ = 9.105; *P* = 0.01]; *Ruminococcaceae_UCG-002* [one-way ANOVA, *F*_(2, 21)_ = 14.13; *P* < 0.001]; *Ruminococcus_2* [one-way ANOVA, *F*_(2, 21)_ = 14.19; *P* < 0.001]; *Catenibacterium* [Kruskal–Wallis non-parametric test, *H*_(2, 21)_ = 19.65; *P* < 0.001]. Data are presented as means ± SEM (*n* = 8 per group). The data that passed the normality and lognormality test were analyzed by one-way ANOVA followed by Tukey's *post hoc* test. Otherwise, Kruskal–Wallis non-parametric test followed by Dunn's *post hoc* test was used. *P < 0.05, **P < 0.01, ***P < 0.001 compared with Sham. ^**#**^*P* < 0.05 compared with CD. CD, cognitive dysfunction; NCD, non-cognitive dysfunction; N.S., not significant.

The 16S rRNA sequencing was performed to explore the alterations in fecal samples from the Sham, CD, and NCD groups. Simpson's Index, an indicator of the α-diversity of gut microbiota, was significantly increased in the CD and NCD groups compared to the Sham group, suggesting lower diversity of species and bacteria after SNI surgery (Figure 2A). Notably, there was no significant change between the CD and NCD groups with regard to the Simpson's Index. Then, the principal component analysis (PCA) and the principal coordinate analysis (PCoA) were used to evaluate the β-diversity, which reflects the similarity and dissimilarity of sample community composition. According to the obtained results, the Sham group clustered far away from the CD and NCD groups, while there was no significant difference between the CD and NCD groups (Figures 2B,C). The heatmap of the gut microbiota composition at different levels exhibited specific differences among the three groups. Figure 2D shows the top 15 dominant microflora in samples at the phylum and genus level, whereas Figures 3A,B show the top 10 significantly differential microbes among the three groups at phylum and genus levels, respectively.

Then, the linear discriminant analysis (LDA) combined with effect size measurements (LEfSe) were used to identify the differentially abundant taxa in the Sham, CD, and NCD groups. Gut bacteria that were enriched in the CD group (and thus depleted in the Sham and NCD groups) included *Proteus* (a member in the *Proteobacteria* phylum) and *Actinobacteria*. Six dominant bacteria were present in the Sham and NCD groups, respectively. In contrast, *Bacteroidaceae, Bacteroides, Citrobacter, Hungatella, Clostridioides*, and *Uncultured_bacterium* were higher in the Sham group as compared to the CD and NCD groups. Interestingly, most of the abundant taxa in the Sham and NCD groups were from *Firmicutes* and *Bacteroidetes* phyla, with the exception of *Citrobacter* and *Uncultured_bacterium* (Figure 3C).

Furthermore, the results revealed that a total of five gut bacteria were significantly increased in the CD mice compared to those in the Sham and NCD groups (Figures 3D–H). It is worth noting that *Nonomuraea*, and *Libanicoccus* were only detected in CD mice, suggesting their promising diagnostic role in patients with CD comorbid with chronic pain, although further studies are required for validation.

### Alterations in Plasma Metabolic Profile Among the Sham, CD, and NCD Mice

Untargeted LC-MS metabolomic profiling identified 101 different metabolites with variable importance in the projection (VIP) scores larger than 1 and *P* < 0.05 as the cutoff. To elucidate on the roles of the differential metabolites, we queried and filtered them in the Human Metabolome Database (HMDB), the Small Molecule Pathway Database (SMPDB), and the Kyoto Encyclopedia of Genes and Genomes (KEGG) database. Notably, only 61 of these 101 metabolites had tentative IDs in the abovementioned databases (details listed in Supplementary Table 2).

**Figure 4:**
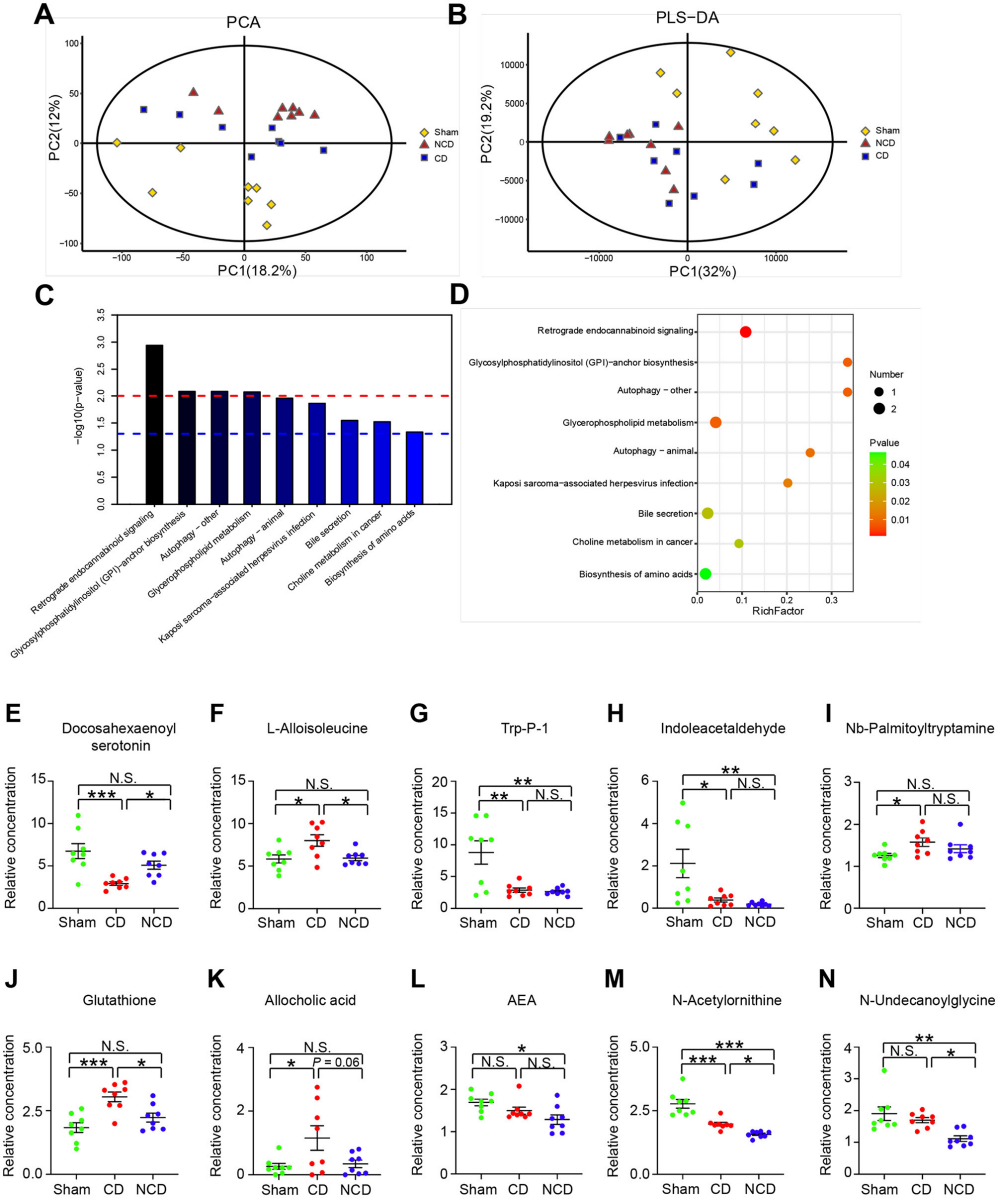
Metabolic profiling analysis among the groups. **(A)** Principal component analysis (PCA). **(B)** The partial least squares discriminant analysis (PLS-DA) of plasma metabolites. **(C)** The Kyoto Encyclopedia of Genes and Genomes (KEGG) pathway analysis of differentially expressed metabolites. Y-axis shows the *P*-value of a particular pathway. **(D)** Enriched KEGG pathways in plasma metabolites. Rich factor indicates the ratio of differentially expressed metabolites to the total metabolites number in a certain pathway. **(E)** Docosahexaenoyl serotonin [one-way ANOVA; *F*_(2, 21)_ = 10.3, *P* < 0.001]; **(F)** L-Alloisoleucine [one-way ANOVA; *F*_(2, 21)_ = 5.84, *P* = 0.009]; **(G)** Trp-P-1 [one-way ANOVA; *F*_(2, 21)_ = 10.49, *P* < 0.001]; **(H)** Indoleacetaldehyde [one-way ANOVA; *F*_(2, 21)_ = 7.259, *P* = 0.004]; **(I)** Nb-Palmitoyltryptamine [one-way ANOVA; *F*_(2, 21)_ = 3.57, *P* = 0.05]; **(J)** Glutathione [one-way ANOVA; *F*_(2, 21)_ = 10.81, *P* < 0.001]; **(K)** Allocholic acid [one-way ANOVA; *F*_(2, 21)_ = 4.24, *P* = 0.028]; **(L)** N-Arachidonoylethanolamine [one-way ANOVA; *F*_(2, 21)_ = 8.015, *P* = 0.018]; **(M)** N-Acetylornithine [one-way ANOVA; *F*_(2, 21)_ = 30.86, *P* < 0.001]; **(N)** N-Undecanoylglycine [one-way ANOVA; *F*_(2, 21)_ = 8.476, *P* = 0.002]. Data are presented as means ± SEM (*n* = 8 per group) and analyzed by one-way ANOVA followed by Tukey's *post hoc* test. *P < 0.05, **P < 0.01, ***P < 0.001. CD, cognitive dysfunction; NCD, non-cognitive dysfunction; N.S., not significant.

The PCA results revealed that the metabolomic profiles differed among the Sham, CD, and NCD groups (Figure 4A), suggesting a pivotal role of plasma metabolites in CNP-induced CD. However, the partial least squares discriminant analysis (PLS-DA) failed to separate the CD group from the NCD group completely (Figure 4B). Then, the KEGG pathway enrichment analysis was performed to explore the functions of the changed metabolites in CNP-induced CD mice. Results revealed that the most enriched KEGG pathways were “Retrograde eCB signaling,” “Glycosylphosphatidylinositol (GPI)-anchor biosynthesis,” “Autophagy-other,” and “Glycerophospholipid metabolism” (Figures 4C,D). These enriched pathways provided novel insights into the pathogenic mechanism of CNP-induced CD, which should be the subject of future research.

Metabolites involved in the retrograde eCB signaling pathway included Anandamide (20: l, n-9)/N-arachidonoylethanolamine (AEA), N-acetylornithine, and N-undecanoylglycine (Figures 4L–N). The relative abundance of the three eCB metabolites presented a downward trend in both CD and NCD mice, suggesting that chronic pain had a greater effect on the plasma metabolites compared with cognitive disorder. With the exception of Nb-palmitoyltryptamine, tryptophan-related metabolites, such as docosahexaenoyl serotonin (DHA-5-HT), Trp-P-1, and indoleacetaldehyde, were significantly decreased in mice with CD (Figures 4E,G–I). In contrast, the relative abundances of L-alloisoleucine, glutathione, and allocholic acid were significantly increased in the CD group compared to the Sham and NCD groups (Figures 4F,J,K).

### Correlations Between Gut Microbiota and Plasma Metabolites

**Figure 5:**
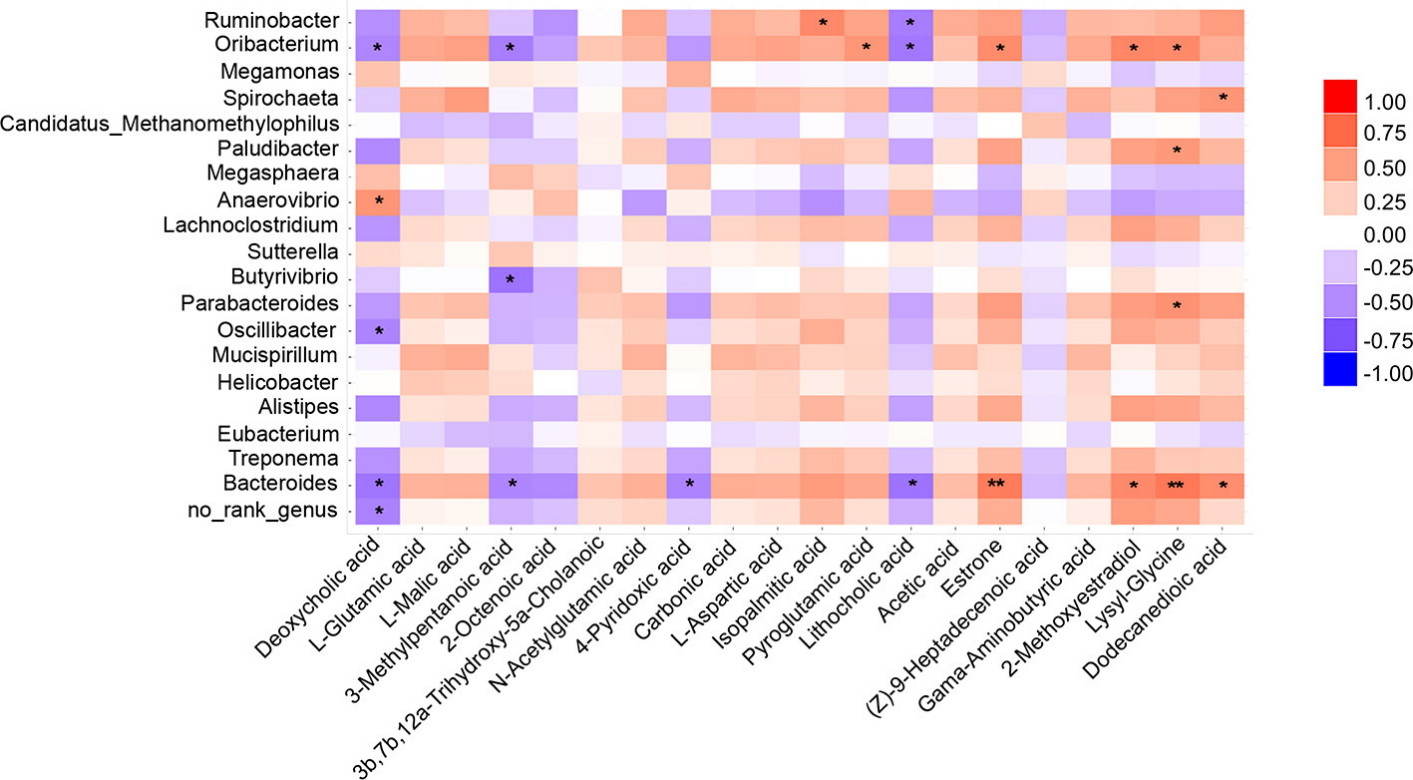
Correlations between plasma metabolite and gut microbiota. Heatmap indicated that some microbes were positively correlated with plasma metabolites and some were negatively correlated with metabolites. *P < 0.05, **P < 0.01.

Spearman's correlation coefficient was applied to evaluate the connection between gut microbes and plasma metabolites (Figure 5). Results showed that *Oribacterium* and *Bacteroides* were strongly correlated with pain-associated metabolites. For example, *Oribacterium* was significantly positively correlated with pyroglutamic acid, estrone, 2-methoxyestradiol, and lysyl-glycine. Significant negative correlations were found with deoxycholic acid, 3-methylpentanoic acid, and lithocholic acid. Interestingly, the same correlation was found between *Bacteroides* and the abovementioned metabolites. In addition, deoxycholic acid was negatively correlated with most microbes, such as *Oribacterium, Oscillibacter*, and *Bacteroides*. However, these microbes were positively correlated with the level of lysyl-glycine. Deoxycholic acid and lysyl-glycine are bile acids and dipeptides, respectively. Moreover, they have been reported to exert physiological or cell-signaling effects on the microbiota-gut-brain axis (Li et al., 2020; Ahn et al., 2021). Collectively, these results suggest that the decreased *Bacteroides*- and *Oribacterium*-related deoxycholic acid accumulation, as well as lysyl-glycine deficiency, might undermine chronic pain-induced CD.

### Effects of FMT on Nociception and Reference Memory in pGF Mice

**Figure 6:**
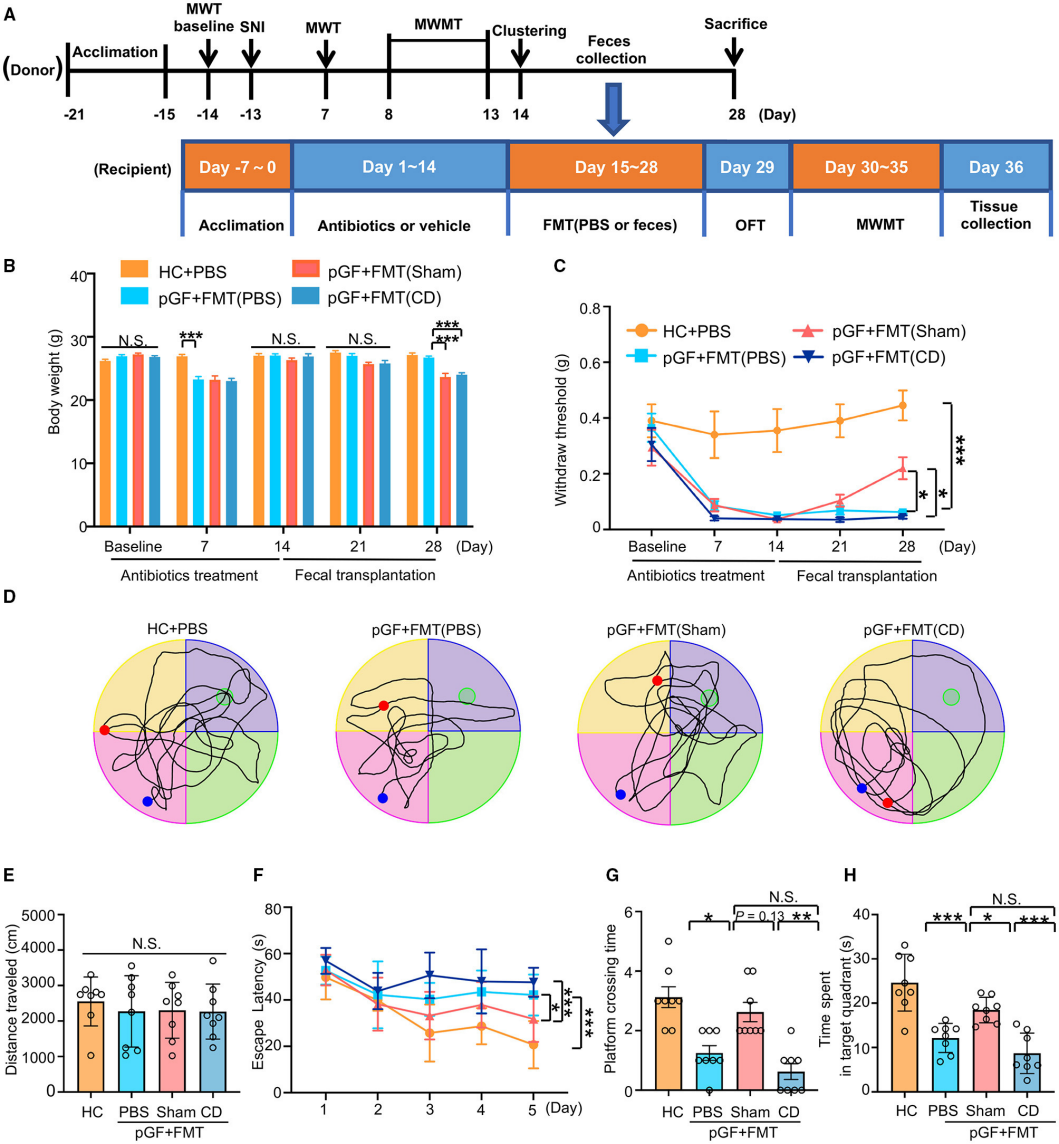
Fecal microbiota transplantation (FMT) from mice of Sham reversed nociception and cognitive impairment in pseudo-germ-free (pGF) mice. **(A)** Experiment schedule of the fecal microbiota donor and receptor mice. To ensure the vitality of the intestinal flora, 1 g fresh feces were collected from the donor mice and then immediately dissolved in 10 ml phosphate-buffered saline (PBS) to prepare suspension for later FMT. For the donor mice, 10-month-old C57 mice were acclimated to the environment from day 21 to day 15 and performed SNI on day 13. MWMT was carried out from day 8 to day 13. After clustering into SNI with CD or NCD group, feces were collected before the FMT from day 15 to day 28. Donor mice were sacrificed after feces collection on day 28. For the receptor mice, 2-month-old C57 mice were treated with broad-spectrum antibiotics in drinking water for 14 consecutive days (day 1 to day 14). Subsequently, mice were treated with 0.2 ml PBS or fecal microbiota suspension obtained from Sham and CD group mice by oral gavage once a day for 14 consecutive days (day 15 to day 28). MWT and body weight were measured on day 1 (baseline), 7, 14, 21, and 28 after SNI. The collection of tissues was performed after MWMT on day 35 after SNI. **(B)** Body weight [two-way RM ANOVA; Time: *F*_(4, 140)_ = 41.8, *P* < 0.001; Group: *F*_(3, 140)_ = 24.76, *P* < 0.001; Interaction: *F*_(12, 140)_ = 8.544, *P* < 0.001]. **(C)** MWT [two-way RM ANOVA; Time: *F*_(4, 140)_ =17.19, *P* < 0.0001; Group: *F*_(3, 140)_ = 48.48, *P* < 0.0001; Interaction: *F*_(12, 140)_ = 2.487, *P* = 0.006]. **(D)** The graphs of the representative traces of control, pGF mice treated with PBS, or gut microbiota from the Sham or CD group mice in the probe test. **(E)** OFT [one-way ANOVA; *F*_(3, 28)_ = 0.2, *P* = 0.88]. **(F)** MWMT [two-way RM ANOVA; Time: *F*_(4, 140)_ = 16.55, *P* < 0.001; Group: *F*_(3, 140)_ = 20.45, *P* < 0.001; Interaction: *F*_(12, 140)_ = 2.152, *P* = 0.017]. **(G)** Platform crossing times [Kruskal–Wallis non-parametric test; *H*_(3, 28)_ = 21.1, *P* = 0.0001]. **(H)** Time spent in target quadrant [one-way ANOVA; *F*_(3, 28)_ = 19.59, *P* < 0.0001]. Data are presented as means ± SEM (*n* = 8 per group) and analyzed by one-way ANOVA or two-way ANOVA followed by Tukey's *post hoc* test or Kruskal–Wallis non-parametric test followed by Dunn's *post hoc* test. *P < 0.05, **P < 0.01, ***P < 0.001. CD, cognitive dysfunction; FMT, fecal microbiota transplantation; HC, healthy control; MWMT, Morris water maze test; MWT, mechanical withdraw threshold; NCD, non-cognitive dysfunction; N.S., not significant; OFT, open field test; pGF, pseudo-germ-free mice; SNI, spared nerve injury.

To investigate whether changes in spatial learning and memory performance in the SNI mice were caused by dysbiosis, pGF mice were established by the oral administration of broad-spectrum antibiotics at large doses for 14 consecutive days. After treatment with antibiotics, gut microbiota from the Sham or CD mice were transplanted into pGF mice for 2 weeks (Figure 6A). The body weight of pGF mice was significantly lower 1 week after antibiotics treatment compared to the control mice but returned to baseline by the end of the antibiotics treatment (day 14). There was no significant difference in body weight among the control, pGF mice receiving PBS, Sham, and CD mice on 21st-day post-antibiotics treatment. However, body weight was significantly lower in the mice receiving Sham and CD fecal transplantation than in the vehicle control mice on the 28th-day posttreatment (Figure 6B). These findings suggested that gut microbiota might influence the metabolism in the host mice. However, food intake during antibiotic treatment should be noticed. There was no difference in MWT among the groups at baseline, but the repeated administration of antibiotics undermined the MWT scores. Interestingly, the FMT from the Sham mice, but not from the CD group mice, significantly restored the allodynia phenotype caused by antibiotics treatment (Figure 6C).

Changes in the total distance traveled in OFT were not different among the four groups before the MWMT (Figure 6E). The representative trace graphs of each group in the probe trial were presented in Figure 6D. Spatial learning and memory performances (including escape latency, platform crossing times, and time spent in the target quadrant) were worse in the PBS group compared to the control group. Furthermore, the transplantation of gut microbiota from the Sham mice, but not from the CD mice, effectively improved the poor performance in the pGF mice (Figures 6F–H). Overall, these results suggest that the behavioral performance was influenced by host microbiota composition.

### Fecal Microbiota From the Sham Mice Normalized PFC *Cnr1, Cnr2*, and *Htr1a* MRNA Expression Caused by Antibiotic Treatment

**Figure 7:**
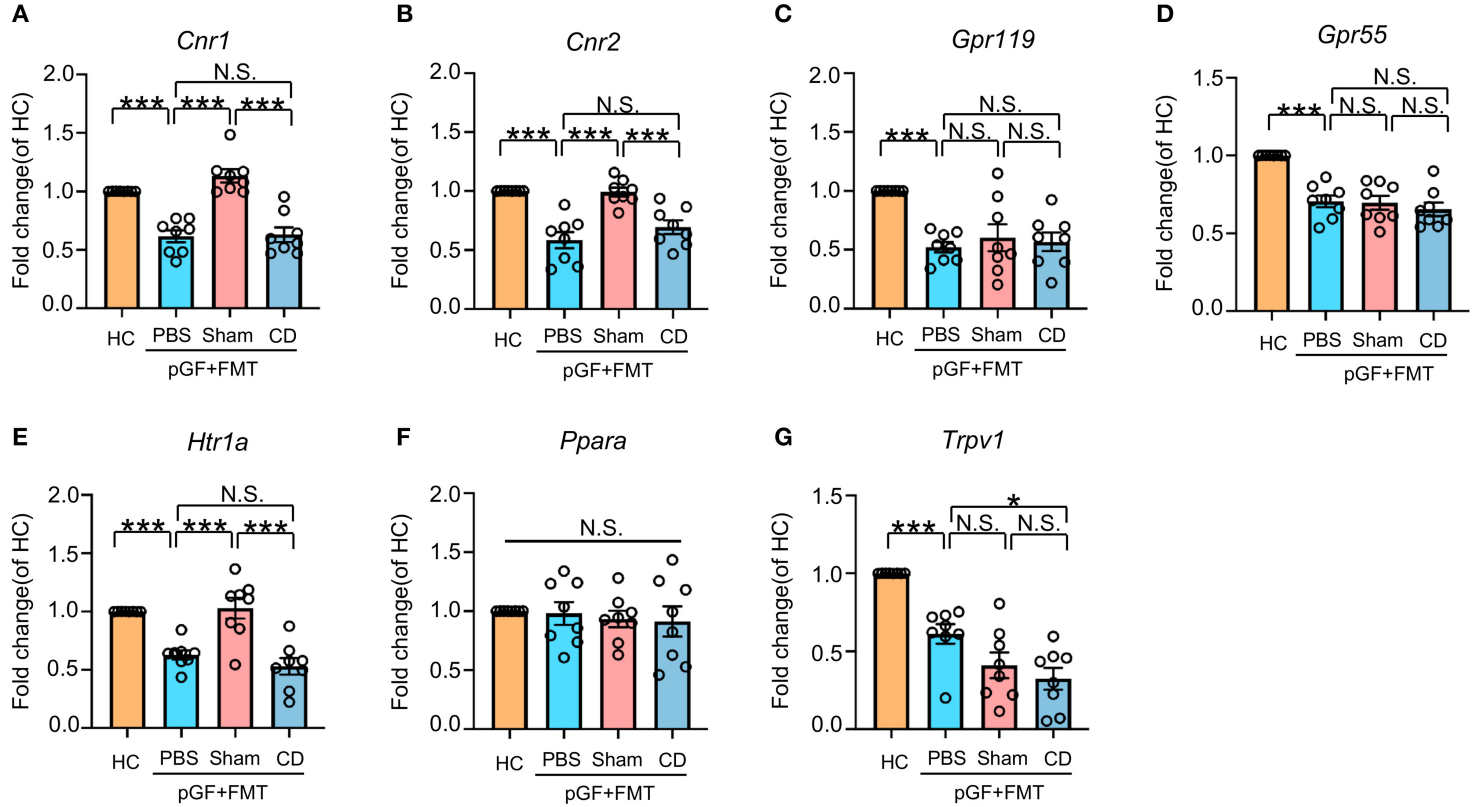
Effects of fecal microbiota transplantation from mice in Sham and CD groups on endocannabinoid (eCB) receptors in pGF mice. **(A)** *Cnr1* [one-way ANOVA; *F*_(2, 21)_ = 28.23, *P* < 0.001]. **(B)** *Cnr2* [one-way ANOVA; *F*_(2, 21)_ = 18.87, *P* < 0.001]. **(C)** *Gpr119* [one-way ANOVA; *F*_(2, 21)_ = 9.10, *P* < 0.001]. **(D)** *Gpr55* [one-way ANOVA; *F*_(2, 21)_ = 19.09, *P* < 0.001]. **(E)** *Htr1a* [one-way ANOVA; *F*_(2, 21)_ = 17.74, *P* < 0.001]. **(F)** *Ppara* [one-way ANOVA; *F*_(2, 21)_ = 0.21, *P* > 0.05]. **(G)** *Trpv1* [one-way ANOVA; *F*_(2, 21)_ = 23.48, *P* < 0.001]. Data are presented as means ± SEM (*n* = 8 per group) and analyzed by one-way ANOVA followed by Tukey's *post hoc* test. *P < 0.05, **P < 0.01, ***P < 0.001. CD, cognitive dysfunction; FMT, fecal microbiota transplantation; HC, healthy control; N.S., not significant; pGF, pseudo-germ-free mice.

To explore the role of eCB signaling in the interaction of dysbiosis and cognition as well as nociception, we measured the mRNA levels of classic cannabinoid receptors (*cn1r* and *cn2r*) and non-cannabinoid receptors, such as peroxisome proliferator-activated receptor alpha (*ppara*), G protein-coupled receptor (GPR) 55 (*gpr55*), *gpr119*, transient receptor potential action channel, subfamily V, member 1 (*trpv1*), and serotonin_1A_ receptor (*htr1a*). It should be noted that these receptors are activated directly or indirectly by eCB, including AEA, 2-arachidonylglycerol (2-AG), N-acylserotonins, and some N-acylethanolamines. Results showed that the mRNA expression of these receptors, with the exception of *ppara*, was reduced in antibiotic-treated mice. However, only *cnr1, cnr2*, and *htr1a* mRNA levels were rescued after receiving bacterial transplantation from the Sham group mice (Figures 7A–G). Therefore, it is likely that the eCB signaling throughout the body serves as a mediator in the cross talk between intestinal bacteria and behavioral performance in the development of chronic pain and CD.

## Discussion

Evidence suggests that CD is one of the most common comorbidities of chronic pain, especially in CNP (Chou et al., 2007; Landro et al., 2013). In this study, SNI mice were clustered into CD (54.8%, 17/31) and NCD (45.2%, 14/31) phenotypes using the hierarchical cluster analysis according to the MWMT performance indices, while mice in CD and NCD groups suffered identical nociception. Compared to the NCD mice, the CD mice exhibited poor learning and memory ability, suggesting that the hierarchical cluster analysis is an effective approach to discriminate those without CD in mice after nerve injury. Consistent with our previous report (Yang et al., 2019), the 16S rRNA diversity analysis showed a consistent decrease in the richness and evenness of gut bacteria in SNI mice when compared with the Sham group, suggesting that alteration in gut microbiota was mainly influenced by chronic pain compared to cognitive disorders. However, the PCA results of plasma metabolites could separate the CD group from the NCD group. Collectively, it is likely that plasma metabolites play a pivotal role in these behavioral abnormalities of cognitive disorder after SNI when the structural dysbiosis of gut microbiota occurred.

Previous studies have demonstrated that some bacterial members from the four phyla (*Firmicutes, Bacteroidetes, Proteobacteria*, and *Actinobacteria*), such as *Bacteroides, Lachnospiraceae*, and *Enterobacteriaceae*, modulate chronic pain and cognitive disorders (Gareau, 2016; Proctor et al., 2017; Li et al., 2020; Chen et al., 2021). In this study, we observed that the relative abundance of *Actinobacteria* was higher in CD than in Sham and NCD mice. Moreover, LDA results indicated that *Actinobacteria* was one of the most important features classifying CD phenotypes in SNI mice. It has been reported that *Actinobacteria* was increased in interstitial cystitis/bladder pain syndrome but decreased in fibromyalgia (Braundmeier-Fleming et al., 2016; Minerbi et al., 2019). In addition, the relative abundance of *Actinobacteria* was increased in patients with Alzheimer's disease (AD) but decreased in triple-transgenic (3xTg) AD mouse model (Zhuang et al., 2018; Bello-Medina et al., 2021). This inconsistency may be attributed to the fact that both potential pathogens and probiotics were contained in the *Actinobacteria* phylum. Collectively, these results suggest that abnormal outgrowth of *Actinobacteria* might contribute to the pathogenesis of CNP and its associated cognitive deficits.

Among the top 10 most significantly differential genera, most of them (*n* = 8) showed consistent changes in the NCD and CD phenotypes, suggesting their important roles in CNP but not in cognitive deficits. Only *Proteus* (genus of *Proteobacteria*) and *Bifidobacterium* (genus of *Actinobacteria*) were associated with CNP-associated cognitive deficits. *Proteus* belongs to the *Enterobacteriaceae* family, which is a hallmark of dysbiosis in the gut (Rivera-Chavez et al., 2017). Notably, species in the *Proteus* are potentially pathogenic bacteria because they may exert pro-inflammatory effects in a host by producing lipopolysaccharide and flagellin proteins (Hamilton et al., 2018). Moreover, the LDA identified that *Proteus* was another gut flora feature that can be used to classify CD phenotype. Therefore, the enrichment of *Proteus* might have contributed to CNP-associated cognitive deficits. Mounting studies have revealed the protective roles of *Bifidobacterium* in various diseases *via* modulating the immune response, glycan metabolism of host, and microbiota-derived metabolites (e.g., short-chain fatty acids) (Turroni et al., 2016; Ruiz et al., 2017). Therefore, the strains of *Bifidobacterium* are widely used as probiotics. These benefits were also observed in studies involving chronic pain or cognitive impairment (Pokusaeva et al., 2017; El-Atawneh et al., 2019). *Bifidobacterium* could modulate visceral pain *via* the enzymatic decarboxylation of glutamate, which induced the production of γ-aminobutyric acid, an important neurotransmitter (Pokusaeva et al., 2017). Moreover, some strains showed therapeutic potential for age-related cognitive impairment, especially AD (El-Atawneh et al., 2019; Zhu et al., 2021), which indicated that *Bifidobacterium* might be beneficial for CNP-associated cognitive deficits. However, in this study, we observed a higher abundance of *Bifidobacterium* in the CD phenotype compared with the other two groups. This is consistent with a few studies that also found increased occurrences of *Bifidobacterium* in several neuropsychiatric disorders (Chung et al., 2019; Ling et al., 2020). In some cases, *Bifidobacterium* species were even regarded as potential pathogens (Pathak et al., 2014). These results made it difficult to determine whether the increase of *Bifidobacterium* was a detrimental factor in SNI-associated cognitive deficits or just an adaptive/compensatory change in the dysbiosis. Therefore, further studies should be conducted to explore the different roles of *Bifidobacterium* species in CNP-associated cognitive deficits. Collectively, these findings suggest that an increased abundance of *Actinobacteria, Proteus*, and *Bifidobacterium* might be the important microbial signatures in CNP-associated cognitive deficits.

Non-targeted plasma metabolic profile was evaluated by LC-MS, and a total of 101 differential metabolites were obtained. The KEGG functional analysis showed that these metabolites were enriched in retrograde eCB signaling, autophagy, bile secretion, and lipids or amino acid biosynthesis and metabolism. Among them, alterations in the retrograde eCB signaling pathway were the most evident. The eCBs are lipid molecules and act as retrograde neurotransmitters that bind cannabinoid receptors and modulate synaptic efficacy (Mulder et al., 2011). The eCB signaling, including two components, namely, AEA and 2-AG, is involved in pain, emotion, stress, and cognition processes (Russo et al., 2018; Mecca et al., 2021; Rea et al., 2021). In this study, the plasma concentrations of AEA in SNI mice were significantly decreased, indicating its potential role in improving SNI-induced neuropathic pain. AEA modulates nociception in the peripheral nociceptors and CNS *via* cannabinoid receptors, TRPV1 channels, and, perhaps, GPR55 (Naz, 1987; Malek et al., 2014). Importantly, AEA is produced to exert anti-inflammatory and neuroprotective effects after nerve injury. However, various studies using different models consistently reported that increases, no changes, or decreases of AEA levels in the spinal cord might be present in chronic pain condition (Malek et al., 2014; Mecca et al., 2021), which were attributed to disparities in activities of receptors, synthesis, and degradation pathways. Various long-chain fatty acid amides, N-acylated amino acids/amines, and their congeners have been reported to exert eCB-like effects on pain and other neurological processes (Di Marzo, 2020). In this study, we established that several plasma fatty acids amides (e.g., N-stearoyl valine) and N-acylated amino acids (e.g., N-Acetylornithine) were significantly decreased in the plasma of SNI mice, consistent with AEA changes, partly due to the fact that this expanded eCB shared inactivating enzymes with AEA (Di Marzo, 2020). With regard to cognitive function, studies have reported that elevated AEA plasma levels in several cognitive disorders and that the pharmacological elevation of AEA may impair memory (Basavarajappa et al., 2014; Cristino et al., 2020). Even though its effects on cognition have not been conclusively determined, AEA elevation induces neuronal excitotoxicity by the long-term activation of the CB1 receptor (Cristino et al., 2020). Consistently, we found that the plasma levels of AEA and the expanded eCB were elevated in the CD phenotype, compared with the NCD phenotype. These findings indicate that, in chronic conditions, elevated AEA and other eCB may contribute to CNP-associated cognitive deficits although they have analgesic properties. Nonetheless, further study is needed to evaluate the role of receptors and metabolic routes of eCB in the comorbidity of CD with CNP.

Of the 61 different metabolites, most are mainly associated with the biosynthesis and metabolism of amino acids and lipids. Amino acids-derived metabolites include N-acyl-amino acids, dipeptides, branched amino acids, glutathione, and several tryptophan catabolites. Glutathione, an important antioxidant agent in the host, was significantly elevated in CD phenotypes, indicating the scavenging of excess reactive oxygen species when CNP-associated cognitive deficits occurred. The levels of four tryptophan catabolites, such as DHA-5-HT, Trp-P-1, indoleacetaldehyde, and Nb-palmitoyltryptamine, were found to be changed. DHA-5-HT is derived from the conjugation of a neurotransmitter (serotonin) and an omega-3 polyunsaturated fatty acid, docosahexaenoyl acid (DHA). Previous studies reported that DHA-5-HT exerted anti-inflammatory effects by inhibiting the interleukin (IL)-23-IL-17 signaling cascade (Poland et al., 2016; Wang et al., 2017). Moreover, it was shown to protect against glutamate-induced cytotoxicity *in vitro* by activating antioxidant enzymes in the CNS (Jin et al., 2014). In this study, DHA-5-HT levels were significantly suppressed in CD phenotypes, indicating the potential disorders of immunomodulation and oxidative stress in CNP-related cognitive deficits. The other three tryptophan catabolites are indole derivatives, which play important roles in the microbiota-gut-brain axis (Roager and Licht, 2018). Compared to DHA-5-HT, their changes were mainly due to the SNI procedure and were less associated with cognitive impairment. Sphingolipids and the derivatives of glycerophospholipids, such as phosphatidylglycerol (PG), phosphatidylcholine (PC), phosphatidylethanolamine (PE), phosphatidylinositol (PI), and phosphatidylserine (PS), are significantly differential lipid metabolites. They are important constituents of cell membranes and play important roles in cell signaling transduction. In this study, we found that glycerophospholipid metabolism was dysregulated in CNP associated with cognitive impairment. Besides, the levels of many phytochemical metabolites, such as americanin B, apritone, licoricone, and 2,3-di-O-methylellagic acid, were decreased in CNP-associated cognitive deficits (N10–19 in Supplementary Table 2). Most of them are polyphenols, which may regulate oxidative stress by the activation of nuclear factor (erythroid-derived 2)-like 2 (Nrf2) pathway (Qin and Hou, 2016). A few differential metabolites are also associated with glucose (D-glucose, D-galactose) as well as bile acid (allocholic acid) metabolism, suggesting the disturbed energy and bile acid metabolism in mice with cognitive impairment under CNP condition. Overall, it is likely that the lipid and amino acid metabolism disturbances might be associated with cognitive deficits in mice with neuropathic pain.

To investigate the role of gut microbiota composition in CNP-associated cognitive deficits, we constructed pGF mice using an antibiotic mix and FMT of the Sham group and CD phenotype into their gastrointestinal tracts. Diminished microbiota and FMT of CD phenotype models induced hyperalgesia and cognitive deficits. Moreover, FMT in the Sham group improved allodynia (at 1 week) and cognitive performance. Given that the eCB signaling pathway was the most significant one in the KEGG analyses, and its promising therapeutic effects on CNP have been widely validated (Starowicz and Finn, 2017; Guida et al., 2020), we evaluated the expressions of several eCB receptors in PFC. PFC, a crucial region, is associated with pain processing and cognition, due to its close connection with other brain areas, such as the hippocampus, amygdala, and thalamus (Baliki et al., 2006; Ong et al., 2019). Diminished microbiota and FMT in the CD phenotype models suppressed the levels of almost all the eCB system-related receptors, except *ppara*. However, FMT in the Sham group only rescued the expressions of *cn1r, cn2r*, and *htr1a*, indicating that the interaction between gut microbiota and eCB system plays a pivotal role in the pathogenic processes in CNP and its associated cognitive deficits.

Recently, growing evidence suggests that eCB system is an important player during the cross talk between gut microbiota and host (Cani et al., 2014). Gut microbiota has been shown to produce eCBs and many bioactive lipids that structurally and functionally resemble eCBs (Cani et al., 2016; Sharkey and Wiley, 2016). Given that the eCB system plays an important role in energy metabolism and inflammation, it may act as a promising therapeutic target for many neurological diseases (Minichino et al., 2021). In this study, we observed that AEA and many expanded eCBs showed significant decreases in the blood when pain and its associated cognitive deficits occurred. Moreover, the changes of FMT in pGF mice demonstrated that gut microbiota could modulate the expressions of eCB receptors in the CNS. These results indicated that eCB system might act as an important mediator between microbiota and the neurological changes induced by CNP. Gut dysbiosis in CNP may cause the declines of eCB ligands and receptors in the brain, resulting in the disorders of eCB signaling transduction, which may aggravate neuroinflammation and the imbalance of brain energy metabolism, finally present as CD. This potential mechanism based on the microbiota-gut-brain axis should be further validated in CNP and its associated cognitive deficits.

## Conclusions

Different compositions of gut microbiome and plasma metabolome were present in CNP-related cognitive impairment models. Peripheral nerve injury dysregulated gut microbiota composition and related metabolites, resulting in decreased cognitive performance. Furthermore, in CNP-associated cognitive impairment, the increased abundance of *Actinobacteria, Proteus*, and *Bifidobacterium* are the important microbial signatures, while metabolomic signatures might be dominated by disturbances in the eCB system, lipid, and amino acid metabolism.

## Data Availability Statement

The data presented in the study are deposited in the MetaboLights repository, accession number MTBLS3891, www.ebi.ac.uk/metabolights/MTBLS3891.

## Ethics Statement

The animal study was reviewed and approved by Experimental Animal Committee of Tongji Hospital, Tongji Medical College, Huazhong University of Science and Technology.

## Author Contributions

DH, ShaL, and AL designed this study and wrote the manuscript. DH, XW, YW, ZX, and YZ performed the experiments. ShiL and JZ analyzed the data. AL revised the manuscript. AL and JZ funded this study. All authors contributed to this study and approved the final manuscript.

## Funding

This study was supported by grants from the National Natural Science Foundation of China (Nos.: 81703482, 81500931, 82171266, and 8217052617).

## Conflict of Interest

The authors declare that the research was conducted in the absence of any commercial or financial relationships that could be construed as a potential conflict of interest.

## Publisher's Note

All claims expressed in this article are solely those of the authors and do not necessarily represent those of their affiliated organizations, or those of the publisher, the editors and the reviewers. Any product that may be evaluated in this article, or claim that may be made by its manufacturer, is not guaranteed or endorsed by the publisher.
