# Supplementary material for: Gut Microbiome and Plasma Metabolome Signatures in Middle-Aged Mice With Cognitive Dysfunction Induced by Chronic Neuropathic Pain
Source: Front Mol Neurosci. 2022 Jan 4;14:806700. doi: 10.3389/fnmol.2021.806700 (PMC8763791; doi:10.3389/fnmol.2021.806700)
Supplement: Supplementary file 1 [file Data_Sheet_1.docx]

Supplementary Material

# Supplementary table 1

Table 1. Primer sequence of endocannabinoid receptors.

| **Target gene** | **Primer sequence** | |
| --- | --- | --- |
|  | **Forward** | **Reverse** |
| *Gapdh* | AGGTCGGTGAACGGATTTG | TGTAGACCATGTAGTTGAGGTCA |
| *Cnr1* | AAGTCGATCTTAGACGGCCTT | TCCTAATTTGGATGCCATGTCTC |
| *Cnr2* | ACGGTGGCTTGGAGTTCAAC | GCCGGGAGGACAGGATAAT |
| *Gpr55* | CACTAAGGGCTGGGTACAAAAG | GCGGTTCCTCACCAGATACTG |
| *Gpr119* | CTTGCTGTCCTAACCATCCTCA | CCACGCCAATCAAGGTATCAG |
| *Ppara* | AGAGCCCCATCTGTCCTCTC | ACTGGTAGTCTGCAAAACCAAA |
| *Trpv1* | TACTATCGGCCTGTGGAAGG | ATTGAATCCCTCGGAAGAAGAAG |
| *Htr1a* | GACAGGCGGCAACGATACT | CCAAGGAGCCGATGAGATAGTT |

# Supplementary Table 2

Table 2. Metabolites with significant changes among the three groups.

| **No.** | **Metabolites** | **Mean(Sham)** | **Mean(CD)** | **Mean(NCD)** | **P-value** |
| --- | --- | --- | --- | --- | --- |
| N1 | Anandamide (20:l, n-9) | 269038.99 | 249704.38 | 228562.81 | 0.02 |
| N2 | N-Acetylornithine | 27681.98 | 19695.61 | 15694.45 | < 0.001 |
| N3 | 1-(Malonylamino)  cyclopropanecarboxylic acid | 157758.40 | 153674.60 | 143211.18 | 0.001 |
| N4 | N-Undecanoylglycine | 1290289.20 | 1269923.71 | 1211516.89 | 0.002 |
| N5 | N-stearoyl valine | 2706900.87 | 2614139.48 | 2409945.85 | < 0.001 |
| N6 | 1. Arachidonoylglycero   phosphoinositol | 116165.37 | 159010.71 | 223002.41 | 0.002 |
| N7 | Pipericine | 87615.58 | 84283.65 | 73624.77 | 0.002 |
| N8 | L-glycyl-L-hydroxyproline | 1205556.57 | 1276372.97 | 1235930.18 | < 0.001 |
| N9 | 2,7-Anhydro-alpha-N-acetylneuraminic acid | 30691.60 | 17914.22 | 16175.10 | < 0.001 |
| N10 | Americanin B | 72844.30 | 64337.42 | 47523.19 | < 0.001 |
| N11 | Adlupone | 7920.79 | 962.28 | 971.16 | < 0.001 |
| N12 | Apritone | 192719.26 | 175061.67 | 126632.59 | < 0.001 |
| N13 | (3beta,6beta)-Furanoere  mophilane-3,6-diol 6-acetate | 1068292.19 | 1069912.51 | 1011889.03 | 0.03 |
| N14 | N,N-dimethyl-Safingol | 236269.55 | 229446.77 | 212885.54 | < 0.001 |
| N15 | Dihydrocapsaicin | 71156.05 | 19098.60 | 17884.05 | < 0.001 |
| N16 | Licoricone | 234857.07 | 231047.98 | 215654.52 | < 0.001 |
| N17 | 2,3-Di-O-methylellagic acid | 314601.04 | 292211.95 | 275833.73 | < 0.001 |
| N18 | Cinnamic acid | 896764.29 | 1122224.09 | 964531.56 | 0.03 |
| N19 | cis-Caryophyllene | 23004.70 | 19090.27 | 13726.02 | < 0.001 |
| N20 | gamma-Glutamylserine | 2291600.38 | 2584064.92 | 2557599.82 | < 0.001 |
| N21 | Tricosanoylglycine | 994150.65 | 1140362.54 | 1123374.35 | 0.001 |
| N22 | Threoninyl-Glycine | 172982.68 | 200742.45 | 200273.34 | < 0.001 |
| N23 | Proline betaine | 36520.76 | 52447.57 | 62573.84 | < 0.001 |
| N24 | Glycyl-Valine | 25584.73 | 17835.39 | 14636.37 | < 0.001 |
| N25 | L-Alloisoleucine | 584535.11 | 801887.21 | 595817.85 | 0.01 |
| N26 | Aspartyl-Cysteine | 1166121.92 | 954535.71 | 949704.94 | 0.02 |
| N27 | L-alpha-Aspartyl-L-  hydroxyproline | 353795.96 | 354592.91 | 336365.50 | 0.02 |
| N28 | 4-Phospho-N-pantothenoyl  cysteine | 135664.29 | 127486.94 | 115823.80 | < 0.001 |
| N29 | 4-Phosphopantothen  oylcysteine | 269654.70 | 268988.12 | 254901.40 | 0.01 |
| N30 | Glutathione | 18355.61 | 30428.89 | 22297.01 | 0.009 |
| N31 | (E)-2-Butenyl-4-methyl-threonine | 57608.12 | 21734.63 | 18328.98 | < 0.001 |
| N32 | Trp-P-1 | 87987.49 | 28749.77 | 26444.08 | < 0.001 |
| N33 | Indoleacetaldehyde | 21130.08 | 3876.14 | 1914.28 | 0.004 |
| N34 | Nb-Palmitoyltryptamine | 125596.94 | 157558.12 | 141653.67 | 0.05 |
| N35 | PI(O-16:0/14:1(9Z)) | 407229.22 | 401260.27 | 361450.31 | < 0.001 |
| N36 | PG(O-20:0/14:1(9Z)) | 75881.18 | 71237.39 | 61083.50 | < 0.001 |
| N37 | D8'-Merulinic acid A | 400155.58 | 398175.43 | 332407.26 | 0.003 |
| N38 | PS(O-16:0/21:0) | 63837.03 | 14895.78 | 11984.94 | 0.004 |
| N39 | PC(o-22:0/20:4  (8Z,11Z,14Z,17Z)) | 92127.30 | 85638.93 | 73961.69 | 0.003 |
| N40 | PC(O-1:0/O-16:0)[U] | 22513.36 | 86.53 | 33.68 | < 0.001 |
| N41 | PC(14:0/20:2(11Z,14Z)) | 2169880.30 | 1815928.25 | 1481035.54 | 0.04 |
| N42 | PC(20:4(5Z,8Z,11Z,14Z)/  16:0) | 509850.43 | 374022.43 | 237153.79 | 0.05 |
| N43 | PC(22:4(7Z,10Z,13Z,16Z)/  15:0) | 253486.75 | 199551.78 | 135447.18 | 0.02 |
| N44 | PE(20:1(11Z)/22:5(4Z,7Z,  10Z,13Z,16Z)) | 512014.18 | 541981.04 | 431957.54 | 0.05 |
| N45 | PE(22:2(13Z,16Z)/16:1(9Z)) | 655630.94 | 557929.36 | 394267.74 | 0.02 |
| N46 | PE(16:1(9Z)/22:1(13Z)) | 232558.59 | 194411.26 | 136753.37 | 0.05 |
| N47 | Phenyl sulfate | 12617.20 | 28138.12 | 42609.09 | < 0.001 |
| N48 | Docosahexaenoyl Serotonin | 674379.75 | 292421.99 | 508321.27 | 0.01 |
| N49 | cis,cis-Muconic acid | 109872.49 | 144173.35 | 142520.01 | < 0.001 |
| N50 | (4E,8E,10E-d18:3)sphingosine | 22371.73 | 12433.16 | 12226.14 | 0.004 |
| N51 | Melleolide | 69842.27 | 64477.44 | 50895.69 | 0.03 |
| N52 | Kojic acid | 493311.03 | 533908.17 | 511100.59 | 0.01 |
| N53 | 2-(3'-Methylthio)  propylmalic acid | 36728.38 | 40647.77 | 43860.01 | < 0.001 |
| N54 | 2-Ketogulonolactone | 30896.49 | 23034.98 | 20354.91 | < 0.001 |
| N55 | (E)-2-Methylglutaconic acid | 43638.56 | 56366.92 | 57615.48 | 0.003 |
| N56 | Heptadecanoyl carnitine | 43841.21 | 54299.28 | 51099.83 | 0.007 |
| N57 | N-Succinyl-2-amino-6  -ketopimelate | 92478.64 | 110143.69 | 119509.30 | 0.04 |
| N58 | D-Galactose | 248714.52 | 205301.83 | 165565.17 | 0.03 |
| N59 | D-Glucose | 170371.03 | 124083.20 | 122876.37 | 0.01 |
| N60 | Uric acid | 31342.30 | 37295.83 | 56393.18 | 0.007 |
| N61 | Allocholic acid | 2655.84 | 11540.75 | 3444.99 | 0.03 |

CD: cognitive dysfunction; NCD: non-cognitive dysfunction; VIP: variable importance in the projection
